# Health inequalities in incidence of bacteraemias: a national surveillance and data linkage study, England, 2018 to 2022

**DOI:** 10.2807/1560-7917.ES.2025.30.9.2400312

**Published:** 2025-03-06

**Authors:** Andrea Mazzella, Zahin Amin-Chowdhury, Amelia Andrews, Andre Charlett, Colin S Brown, Russell Hope, Dimple Chudasama

**Affiliations:** 1 UK Health Security Agency, London, United Kingdom

**Keywords:** bacteraemia, ethnicity, socioeconomic factors, health inequities, public health surveillance

## Abstract

**Background:**

Health inequalities exist globally, but limited data exist on this topic for bacteraemia.

**Aim:**

In this study we investigated health inequalities surrounding bacteraemia in England, to identify high-risk population groups and areas of intervention.

**Methods:**

We retrospectively analysed English surveillance data between 2018 and 2022 for *Escherichia coli*, *Klebsiella* species, *Pseudomonas aeruginosa*, and both meticillin-sensitive and resistant *Staphylococcus aureus* (MSSA, MRSA) bacteraemia. Crude incidence rates stratified by index of multiple deprivation and ethnic groups were calculated; age-adjusted rate ratios were estimated using negative binomial regression models.

**Results:**

We identified 342,787 bacteraemia cases. Across all pathogens, as the level of deprivation rose, so did the age-adjusted bacteraemia incidence rate ratio. Compared with residents of the 20% least deprived areas of England, residents of the 20% most deprived areas had a 2.68-fold increased bacteraemia rate for MRSA (95% CI: 2.29–3.13) and 1.95-fold for *E. coli* (95% CI: 1.84–2.05), and 15% higher odds of dying within 30 days of any bacteraemia (95% CI: 1.13–1.19). After age adjustment, the incidence of all bacteraemia was higher in the Asian and Black groups compared with the White group: for MRSA, 79% higher in the Asian (95% CI: 1.51–2.10) and 59% higher in the Black (95% CI: 1.29–1.95) groups. The exception was MSSA, whose incidence was highest in the White group.

**Conclusion:**

Disproportionately higher age-adjusted incidence of bacteraemia occurred in deprived areas and ethnic minorities. These disparities are likely multifactorial, possibly including socioeconomic, cultural, and systemic risk factors and different burden of comorbidities. Better understanding these factors can enable targeted interventions.

**Key public health message**

**What did you want to address in this study and why?**

Socioeconomic inequalities exist across infections. We wanted to assess if the incidence of common bacteraemia varies across deprivation level and ethnic groups in England and to quantify any differences. The aim was to identify high-risk population groups and areas for public health interventions. Between 2018 and 2022, we investigated bacteraemia caused by *Escherichia coli*, *Klebsiella* and *Pseudomonas aeruginosa* bacterial species as well as by meticillin-susceptible and resistant *Staphylococcus aureus* (MRSA, MSSA).

**What have we learnt from this study?**

In England, bacteraemia from *E. coli*, *Klebsiella* spp., *P. aeruginosa* and particularly MRSA disproportionately affected people from the Black and Asian ethnic groups, after adjusting for age. In contrast, the White ethnic group had higher incidence of community-onset MSSA bacteraemia. People living in the 20% most deprived areas of England also had higher age-standardised incidence of all bacteraemia (176.7 compared to 91.7 per 100,000 population per year in the 20% least deprived areas), and especially MRSA bacteraemia.

**What are the implications of your findings for public health?**

Public health agencies and health research organisations should consider stratifying their analyses by ethnicity and deprivation to identify and quantify health inequalities. Further studies including qualitative research is required to identify the root causes of these concerning inequalities in the incidence of bacteraemia caused by different pathogens, so that adapted measures can be designed to mitigate them.

## Introduction

Bacteraemia is an acute infection with considerable morbidity and mortality and increased healthcare costs. While certain patient characteristics, including extremes of age, male sex and comorbidities, are associated with a higher risk of developing bacteraemia, there is limited understanding about its wider social determinants [1].

Europe has a large socioeconomically and ethnically diverse population, with England being no exception; recent population statistics observe that the percentage of the population in non-White ethnic groups increased from 14% in 2011 to 18% in 2021 [2]. In the 2021 Census, 81.7% of England and Wales residents identified their broad ethnic group (EG) as White (median age: 43 years), 9.3% as Asian (or Asian British; 32 years), 4.0% as Black (or Black British; 32 years), 2.9% as Mixed (or Multiple; 19 years) and 2.1% as Other (32 years) [3]. Health inequalities exist within England, with evidence suggesting that socioeconomically deprived and minority ethnic groups are disproportionately affected by poorer health outcomes; for example, shorter life expectancies, higher prevalence of certain nosocomial infections in more deprived areas [4,5], increased COVID-19 incidence [6] and mortality [7] among people from South Asian ethnic groups, as well as higher likelihood of long COVID symptoms in minority ethnicities [8]. The coincidence between undesirable health outcomes and deprivation or belonging to an ethnic minority has also been observed in other countries. In Denmark, two studies identified an association between lower socioeconomic status and increased risk of *Staphylococcus aureus* bacteraemia, even after adjusting for comorbidities [9,10]. Studies identified higher incidence of *S. aureus* bacteraemia in the Māori and Pacific Island people in New Zealand [11] and among Indigenous people in Australia [12]. A Dutch prospective cohort study identified substantial inequalities in various health outcomes between ethnic groups, only partly explained by socioeconomic status [13].

Ethnicity and race are social constructs, with varying interpretations in different countries and hold no intrinsic biological significance [14,15]. In certain countries, censuses do not collect or may even prohibit collecting information about people’s ethnicity [16]. However, these factors carry important societal meanings and are linked to health inequalities [17], so it remains important for public health to report on the former, to assess and address the latter [14].

The objective of this study was to analyse the recent epidemiological trends of bacteraemia in England by deprivation and ethnic groups, focussing on the 20% most deprived areas and ethnic minority communities. This can be used to identify population groups whose social characteristics may be associated with an increased risk of bacteraemia, with the aim of informing action to reduce health inequalities and tackle the wider threat of antimicrobial resistance.

## Methods

### Setting and study design

This was a retrospective surveillance and data linkage study, based on surveillance data from England between January 2018 and December 2022. Patients of all ages were included.

The United Kingdom (UK) Health Security Agency conducts continuous national mandatory surveillance of bacteraemia caused by four priority bacteria: three Gram-negative bacteria (*Escherichia coli*, *Klebsiella* species, *Pseudomonas aeruginosa*) and meticillin-sensitive (MSSA) and resistant *S. aureus* (MRSA). All cases from public sector hospitals were included in this study. Cases identified post-mortem are excluded from surveillance.

### Data sources and definitions

Bacteraemia was defined as a blood culture positive for one of the mentioned pathogens. Infections were classified as hospital-onset (HO) if the specimen was collected in an acute hospital on day 3 or later from admission, where date of admission is day 1; community-onset (CO) otherwise. Repeat specimens that were positive for the same pathogen in the subsequent 14 days were excluded [18].

To align with a National Health Service (NHS) England approach to inform action for reducing healthcare inequalities (Core20PLUS5) [19], which identifies populations in the 20% most deprived areas as a target, deprivation was defined as quintile of index of multiple deprivation (IMD) 2019. This is a composite measure which relatively ranks Lower-layer Super Output Areas (LSOA) in England from most to least deprived. All LSOAs in England (each with an average of 1,500 residents) [20] were included in the study. The seven domains that make up the IMD are income, employment, education, health, crime, barriers to housing and services, and living environment.

Each bacteraemia case was deterministically linked via the postcode of residence at the time of infection to an LSOA and its IMD quintile. Unlinked cases were excluded from IMD analyses.

Populations by deprivation level were calculated by summing the LSOA populations for each IMD quintile. We used mid-year population estimates published by the UK Office of National Statistics for years 2018, 2019, and 2020; mid-2020 estimates were carried forward for 2021 and 2022.

Ethnicity is self-stated at the time of hospital admission. Broad EGs were defined according to the Office of National Statistics classification; each group is made up of a series of detailed EGs [2].

Ethnicity data were obtained via data linkage from the NHS Hospital Episode Statistics (HES), covering secondary care activity in NHS hospitals in England [21]. Hospital guidance specifies that ethnicity should be self-reported by patients. For each patient, we captured all recorded ethnicities from all hospital admissions in HES across their lifetime, regardless of whether the admission was related to bacteraemia. When patients had different recorded ethnicities in different hospital admissions, we selected a single ethnicity by using an algorithm developed by the UK Office for Health Improvement and Disparities [22]. Cases were then deterministically linked by NHS number (a unique identifier) and date of birth. Unlinked cases were excluded from ethnicity analyses.

For population demographics, we used Office of National Statistics populations by detailed EG and age group from the 2021 national census, during which ethnicity data were self-reported. We calculated populations by broad EG by summing the detailed EG populations (e.g. we calculated the total ‘White’ EG population by summing the ‘White – British’, ‘White – Irish’ and ‘White – Any other White background’ populations).

Date of death was obtained via data linkage to the NHS Spine (https://digital.nhs.uk/services/spine); records were traced using date of birth and NHS number. Unlinked cases were excluded from the case fatality analyses. Episodes of bacteraemia were de-duplicated within a 30-day window before mortality analysis: if a patient had two episodes caused by the same pathogen and died within a 30-day window, only the later episode was kept, to avoid double counting their death.

### Statistical methods

Data processing, linkage, analysis, and visualisations were conducted using R version 4.3.0 [23].

Stratified incidence rates were calculated and presented as yearly averages per 100,000 population. Assuming an alpha value of 0.05, Poisson 95% confidence intervals (CI) were constructed using the exact method. Direct age standardisation using the 2013 European Standard Population [24] was used to estimate age-standardised incidence rates; their 95% CI were calculated using Byar’s method with Dobson adjustment.

IMD- and ethnicity-stratified incidence rates of HO bacteraemia were calculated using population denominators, as occupied hospital bed-days are not available stratified by IMD or ethnic group.

Age-adjusted rate ratios (aRR) and their 95% CI were estimated using negative binomial regression models (as implemented in R package MASS). The dependent variable was the count of infections in each age and exposure (IMD or ethnicity) group. The exposure group and the 5-year age interval served as categorical covariates, while the natural logarithm of the population in the respective exposure/age group was employed as an offset. Estimates were exponentiated. The reference groups were the 20% least deprived areas (5^th^ quintile), for IMD, and the White group, for ethnicity.

All-cause case fatality rates at 30 days (CFR) were calculated by IMD quintile and by ethnic group; binomial 95% CIs were estimated using the exact method. To control the effect of age, age-adjusted odds ratios (aOR) were estimated, using two logistic regression models: 30-day mortality was the dependent variable, while the covariates were 5-year age interval and either IMD quintile or ethnic group.

We followed the REporting of studies Conducted using Observational Routinely-collected Data (RECORD) reporting guidelines and GUidance for Information about Linking Data sets (GUILD) [25,26].

## Results

### Characteristics

Between 2018 and 2022, there were a total of 342,787 cases of bacteraemia (Table 1), with the majority being *E. coli* (200,530; 58.5%), followed by MSSA (61,257; 17.9%), *Klebsiella* spp. (55,673; 16.2%), *P. aeruginosa* (21,515; 6.3%) and MRSA (3,812; 1.1%). The median age across all pathogens was 73 years (interquartile range (IQR): 59–83), while the age distribution appeared to be younger for *S. aureus* bacteraemias (for MSSA: median 65 years; IQR: 47–79).

**Table 1:** Summary of bacteraemia cases identified during the study, England, January 2018–December 2022 (n = 342,787 cases)

Characteristic	Group	Cases of bacteraemia											
		Overall n = 342,787		Caused by *E. coli* n = 200,530		Caused by *Klebsiella* spp. n = 55,673		Caused by *P. aeruginosa* n = 21,515		Caused by MSSA n = 61,257		Caused by MRSA n = 3,812	
		Number	%^a^	Number	%^a^	Number	%^a^	Number	%^a^	Number	%^a^	Number	%^a^
Sex	Female	152,874	44.6	100,963	50.4	20,675	37.2	7,675	35.7	22,219	36.3	1,342	35.2
	Male	189,728	55.4	99,463	49.6	34,969	62.8	13,832	64.3	38,996	63.7	2,468	64.8
	Unknown	185	N/A	104	N/A	29	N/A	8	N/A	42	N/A	2	N/A
Median age in years (IQR)		73 (59–83)		75 (63–84)		72 (59–81)		72 (58–82)		65 (47–79)		67 (47–81)	
Index of Multiple Deprivation quintile	1^st^ (most deprived)	77,577	23.0	44,166	22.4	12,511	22.9	4,131	19.5	15,743	26.2	1,026	27.6
	2^nd^	71,072	21.1	41,142	20.8	11,915	21.8	4,402	20.8	12,762	21.3	851	22.9
	3^rd^	67,567	20.0	39,724	20.1	11,036	20.2	4,523	21.4	11,576	19.3	708	19.0
	4^th^	63,492	18.8	38,131	19.3	9,984	18.3	4,222	19.9	10,562	17.6	593	16.0
	5^th^ (least deprived)	57,405	17.0	34,391	17.4	9,174	16.8	3,898	18.4	9,403	15.7	539	14.5
	Unknown	5,674	N/A	2,976	N/A	1,053	N/A	339	N/A	1,211	N/A	95	N/A
Ethnic group	Asian	21,025	6.4	11,992	6.2	4,212	7.9	1,271	6.2	3,170	5.4	380	10.5
	Black	9,924	3.0	5,024	2.6	2,237	4.2	902	4.4	1,608	2.7	153	4.2
	Mixed	2,610	0.8	1,302	0.7	504	0.9	192	0.9	575	1.0	37	1.0
	White	293,012	89.3	172,807	90.0	45,993	86.4	18,106	88.0	53,071	90.5	3,035	83.7
	Other	1,628	0.5	944	0.5	308	0.6	103	0.5	250	0.4	23	0.6
	Unknown	14,588	N/A	8,461	N/A	2,419	N/A	941	N/A	2,583	N/A	184	N/A
Onset	Community-onset	261,423	76.3	163,646	81.6	38,015	68.3	13,431	62.4	43,840	71.6	2,491	65.3
	Hospital-onset	81,364	23.7	36,884	18.4	17,658	31.7	8,084	37.6	17,417	28.4	1,321	34.7

*E. coli*: *Escherichia coli*; IQR: interquartile range; MRSA: meticillin-resistant *Staphylococcus aureus*; MSSA: meticillin-susceptible *Staphylococcus aureus*; N/A: not applicable; *P. aeruginosa*: *Pseudomonas aeruginosa*.

^a^ Percentages refer to the total of known values in each column.

Hospital-onset infections (n = 81,364) constituted 23.7% of the total; however, this percentage appeared to be higher for *P. aeruginosa* (8,084; 37.6%), MRSA (1,321; 34.7%), *Klebsiella* spp. (17,658; 31.7%), and MSSA (17,417; 28.4%), and only lower for *E. coli* (36,884; 18.4%).

Combined *S. aureus* results are presented in the Supplementary Tables 1–3, to facilitate comparison with other studies which may not report on MSSA and MRSA separately.

Deprivation linkage was successful in 337,113 of 342,787 cases (98.3%). The remaining cases could not be linked because of various reasons: missing postcode in the data source (3,995; 1.2%), patients residing outside England (1,282; 0.4%), homelessness (186; 0.05%), or because of other reasons (211; 0.06%). These causes were consistent across the pathogens, except for a slightly higher frequency of homelessness observed among *S. aureus* cases (data not shown).

Incidence of bacteraemia appeared higher in increasing levels of deprivation, with 23.0% of bacteraemias in the most deprived areas (n = 77,577) and 17.0% in the least deprived areas (n = 57,405).

Ethnicity linkage was successful in 328,199 of 342,787 cases (95.7%); for the remaining cases, ethnicity was unknown because of linkage failure (6,257; 1.8%), the patient had decided not to state their ethnicity (4,910; 1.4%), the NHS number was unknown (2,882; 0.8%), or because ethnicity was recorded as ‘Not known’ (539; 0.2%). These causes were consistent across the pathogens (data not shown).

Among cases where ethnicity was known, 89.3% occurred in the White EG (n = 293,012), while the Asian EG accounted for 6.4% (n = 21,025), the Black EG for 3.0% (n = 9,924), the Mixed EG for 0.8% (n = 2,610), and the Other EG for 0.5% of cases (n = 1,628). More than 30% of cases from the Black (3,804/9,924; 38.5%), Asian (6,581/21,025; 31.4%), Mixed (817/2,610; 31.6%), Other (506/1,628; 31.4%) EGs lived in the most deprived areas of England, while only 21.8% (63,581/293,012) of cases from the White EG lived in those areas as presented in the Supplementary Table 4.

### Incidence by index of multiple deprivation

**Figure 1:**
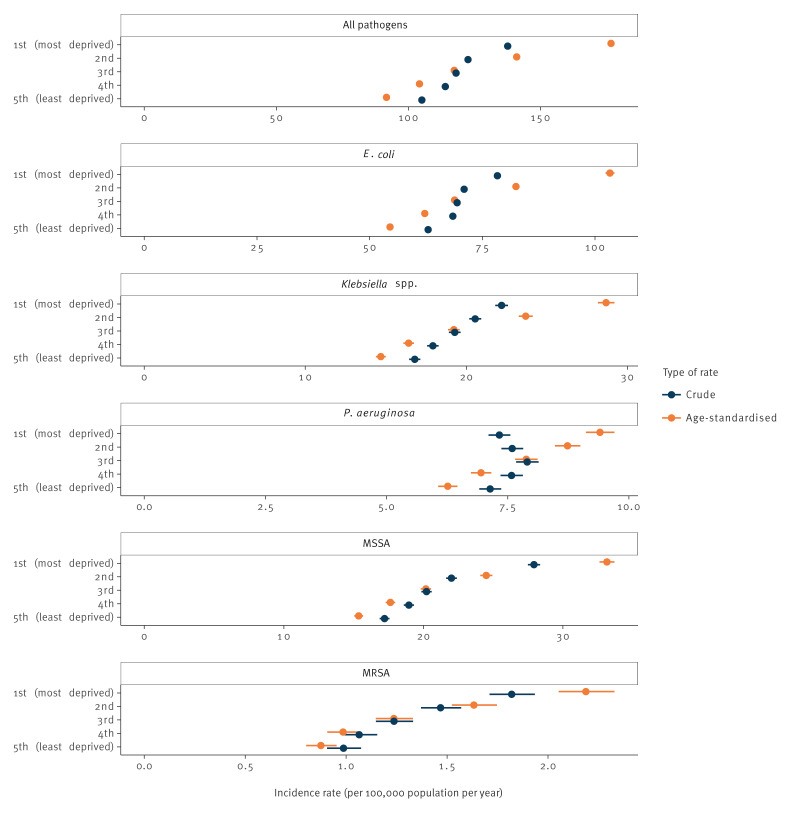
Crude and age-standardised incidence rate of bacteraemia by index of multiple deprivation group, England, January 2018–December 2022 (n = 342,787 cases)

Across the pathogens, except *P. aeruginosa*, as the level of deprivation increased, so did the crude incidence rate of bacteraemia (Figure 1, Table 2). There were 105.1 bacteraemias from any pathogen per 100,000 population per year in the 20% least deprived areas of England, while there were 137.6 per 100,000 in the 20% most deprived areas. After accounting for age, these rates were further apart, respectively 91.7 (95% CI: 91.0–92.5) and 176.7 (95% CI: 175.5–178.0) per 100,000 population per year, with an age-adjusted rate ratio of 2.01 (95% CI: 1.89–2.15) between these two groups.

*E. coli*: *Escherichia coli*; MRSA: meticillin-resistant *Staphylococcus aureus*; MSSA: meticillin-susceptible *Staphylococcus aureus*; *P. aeruginosa*: *Pseudomonas aeruginosa*.

The index of multiple deprivation groups (i.e. quintile) are indicated on the Y axis. The ranges indicate the 95% confidence intervals around the point estimates; some are too narrow to be visible.

**Table 2:** Cases of bacteraemia by pathogen and index of multiple deprivation quintile, England, January 2018–December 2022 (n = 342,787 cases)

Pathogens considered in the study	IMD quintile	Cases	Age^a^	Crude rate^b,c^	Age-standardised rate^b,c^	Age-adjusted rate ratio^b,c^
All	1^st^ (most deprived)	77,577	68 (52–80)	137.6 (136.6–138.6)	176.7 (175.5–178.0)	2.01 (1.89–2.15)
	2^nd^	71,072	72 (57–82)	122.6 (121.7–123.5)	141.0 (140.0–142.1)	1.55 (1.46–1.66)
	3^rd^	67,567	74 (61–83)	118.0 (117.2–118.9)	117.4 (116.5–118.3)	1.29 (1.21–1.37)
	4t^h^	63,492	75 (64–84)	114.0 (113.1–114.9)	104.2 (103.4–105.0)	1.12 (1.05–1.20)
	5^th^ (least deprived)	57,405	76 (65–85)	105.1 (104.2–106.0)	91.7 (91.0–92.5)	Reference
	Unknown	5,674	58 (32–74)	N/A	N/A	N/A
*E. coli*	1^st^ (most deprived)	44,166	72 (58–82)	78.3 (77.6–79.1)	103.3 (102.3–104.3)	1.95 (1.84–2.05)
	2^nd^	41,142	74 (61–83)	71.0 (70.3–71.6)	82.5 (81.7–83.3)	1.52 (1.44–1.60)
	3^rd^	39,724	76 (64–84)	69.4 (68.7–70.1)	68.8 (68.2–69.5)	1.25 (1.18–1.32)
	4^th^	38,131	77 (66–85)	68.5 (67.8–69.1)	62.2 (61.6–62.9)	1.12 (1.06–1.18)
	5^th^ (least deprived)	34,391	78 (67–85)	63.0 (62.3–63.6)	54.5 (53.9–55.1)	Reference
	Unknown	2,976	63 (36–76)	N/A	N/A	N/A
*Klebsiella *spp.	1^st^ (most deprived)	12,511	68 (54–79)	22.2 (21.8–22.6)	28.7 (28.2–29.2)	2.01 (1.88–2.15)
	2^nd^	11,915	70 (57–81)	20.5 (20.2–20.9)	23.7 (23.3–24.1)	1.66 (1.55–1.77)
	3^rd^	11,036	73 (60–82)	19.3 (18.9–19.6)	19.2 (18.9–19.6)	1.33 (1.24–1.42)
	4^th^	9,984	74 (63–83)	17.9 (17.6–18.3)	16.4 (16.1–16.7)	1.12 (1.04–1.20)
	5^th^ (least deprived)	9,174	75 (64–84)	16.8 (16.5–17.1)	14.7 (14.4–15.0)	Reference
	Unknown	1,053	58 (32–72)	N/A	N/A	N/A
*P. aeruginosa*	1^st^ (most deprived)	4,131	68 (53–79)	7.3 (7.1–7.6)	9.4 (9.1–9.7)	1.53 (1.43–1.65)
	2^nd^	4,402	70 (56–81)	7.6 (7.4–7.8)	8.7 (8.5–9.0)	1.40 (1.31–1.50)
	3^rd^	4,523	72 (59–82)	7.9 (7.7–8.1)	7.9 (7.7–8.1)	1.29 (1.20–1.38)
	4^th^	4,222	74 (63–83)	7.6 (7.4–7.8)	6.9 (6.7–7.2)	1.10 (1.02–1.18)
	5^th^ (least deprived)	3,898	75 (64–84)	7.1 (6.9–7.4)	6.3 (6.1–6.5)	Reference
	Unknown	339	56 (30–70)	N/A	N/A	N/A
MSSA	1^st^ (most deprived)	15,743	57 (40–73)	27.9 (27.5–28.4)	33.2 (32.6–33.7)	2.26 (2.04–2.52)
	2^nd^	12,762	63 (46–77)	22.0 (21.6–22.4)	24.5 (24.1–24.9)	1.61 (1.45–1.79)
	3^rd^	11,576	68 (51–80)	20.2 (19.9–20.6)	20.2 (19.8–20.6)	1.33 (1.19–1.48)
	4^th^	10,562	71 (55–82)	19.0 (18.6–19.3)	17.6 (17.3–18.0)	1.16 (1.04–1.29)
	5^th^ (least deprived)	9,403	73 (57–83)	17.2 (16.9–17.6)	15.4 (15.1–15.7)	Reference
	Unknown	1,211	45 (29–65)	N/A	N/A	N/A
MRSA	1^st^ (most deprived)	1,026	57 (41–75)	1.8 (1.7–1.9)	2.2 (2.1–2.3)	2.68 (2.29–3.13)
	2^nd^	851	64 (45–79)	1.5 (1.4–1.6)	1.6 (1.5–1.7)	1.94 (1.66–2.28)
	3^rd^	708	67 (50–81)	1.2 (1.1–1.3)	1.2 (1.1–1.3)	1.47 (1.25–1.73)
	4^th^	593	74 (60–84)	1.1 (1.0–1.2)	1.0 (0.9–1.1)	1.13 (0.96–1.34)
	5^th^ (least deprived)	539	76 (62–85)	1.0 (0.9–1.1)	0.9 (0.8–1.0)	Reference
	Unknown	95	44 (30–62)	N/A	N/A	N/A

*E. coli*: *Escherichia coli*; IMD: index of multiple deprivation; MRSA: meticillin-resistant *Staphylococcus aureus*; MSSA: meticillin-susceptible *Staphylococcus aureus*; N/A: not applicable; *P. aeruginosa*: *Pseudomonas aeruginosa*.

^a^ Median age in years (interquartile range).

^b^ Rates are incidence rates per 100,000 population per year.

^c^ Point estimate (95% confidence interval).

Rate ratios by IMD were highest for *S. aureus* bacteraemia: the most deprived areas of England had 2.68 times higher incidence than the least deprived areas for MRSA (95% CI: 2.29–3.13) and 2.26 times higher for MSSA (95% CI: 2.04–2.52). Disproportionate rates were also seen for Gram-negative bacteraemia, but less conspicuous: 2.01 times higher for *Klebsiella* spp. (95% CI: 1.88–2.15); 1.95 for *E. coli* (95% CI: 1.84–2.05); 1.53 for *P. aeruginosa* (95% CI: 1.43–1.65).

Median age at time of infection appeared to be lower in the most deprived areas than in the least deprived areas, particularly for *S. aureus* bacteraemias (e.g. 57 vs 76 years for MRSA), but also for Gram-negative bacteraemias (e.g. 68 vs 75 years for *Klebsiella* spp.). Of note, cases with unknown IMD quintile tended to be younger than those with known IMD; this was mostly due to neonates often not having a known postcode of residence (data not shown).

### Incidence by ethnic group

The White ethnic group had a higher crude incidence rate than any other group for all bacteraemia except MRSA: across all pathogens, 128.0 (95% CI: 127.5–128.5) per 100,000 population per year, compared with 83.3 (95% CI: 81.7–85.0) in the Black and 77.5 (95% CI: 76.4–78.5) in the Asian group. However, this was substantially influenced by age, as the median age at time of infection appeared to be substantially higher in the White EG.

**Figure 2:**
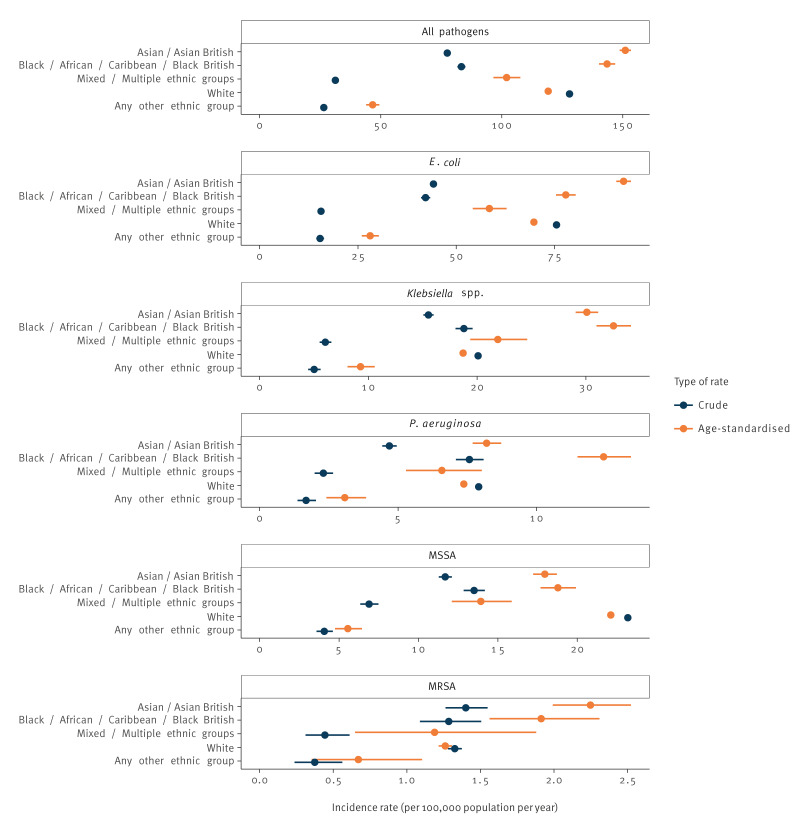
Crude and age-standardised incidence of bacteraemia by ethnic group, England, January 2018–December 2022 (n = 342,787 cases)

The age-adjusted incidence rate of any bacteraemia was 27% higher in the Black EG (aRR: 1.27; 95% CI: 1.18–1.37) and 19% higher in the Asian EG (aRR: 1.19; 95% CI: 1.11–1.27), compared with the White EG (Figure 2, Table 3). Differences were particularly pronounced for bacteraemia caused by MRSA and Gram-negative bacteria. The age-adjusted incidence rates of MRSA in the Asian and Black EG were respectively 1.79 and 1.59 times higher than in the White EG (95% CI, respectively: 1.51–2.10 and 1.29–1.95). Age-adjusted rate ratios of *Klebsiella* spp. bacteraemia were higher in the Black (1.84; 95% CI: 1.76–1.92) and Asian (1.58; 95% CI: 1.53–1.63) groups compared with the White group; slightly lower rate ratios were seen for *E. coli*. For *P. aeruginosa* bacteraemia, the Black EG had the highest age-adjusted rate ratio (1.84; 95% CI: 1.72–1.97) compared with the White group. Conversely, the White EG had the highest age-adjusted rate of MSSA bacteraemia: this was 25% higher than in the Asian (aRR: 0.80; 95% CI: 0.72–0.89) and 14% higher than in the Black EG (aRR: 0.88; 95% CI: 0.79–0.99).

The ranges indicate the 95% confidence intervals around the point estimate; some are too narrow to be visible.

**Table 3:** Cases of bacteraemia by pathogen and ethnic group, England, January 2018–December 2022 (n = 342,787 cases)

Pathogens considered in the study	Ethnic group	Cases	Age^a^	Crude rate^b,c^	Age-standardised rate^b,c^	Age-adjusted rate ratio^b,c^
All	Asian	21,025	64 (44–77)	77.5 (76.4–78.5)	151.0 (148.7–153.4)	1.19 (1.11–1.27)
	Black	9,924	58 (42–74)	83.3 (81.7–85.0)	143.5 (140.2–146.9)	1.27 (1.18–1.37)
	Mixed	2,610	48 (21–68)	31.3 (30.1–32.5)	102.0 (96.5–107.6)	0.79 (0.73–0.85)
	White	293,012	74 (61–83)	128.0 (127.5–128.5)	119.2 (118.8–119.6)	Reference
	Other	1,628	55 (36–72)	26.5 (25.2–27.8)	46.7 (44.0–49.4)	0.43 (0.4–0.47)
	Unknown	14,588	67 (47–79)	N/A	N/A	N/A
*E. coli*	Asian	11,992	67 (49–79)	44.2 (43.4–45.0)	92.5 (90.7–94.4)	1.25 (1.14–1.36)
	Black	5,024	61 (45–78)	42.2 (41.0–43.4)	77.8 (75.4–80.4)	1.21 (1.11–1.33)
	Mixed	1,302	53 (30–72)	15.6 (14.8–16.5)	58.4 (54.2–62.8)	0.78 (0.71–0.86)
	White	172,807	76 (65–84)	75.5 (75.1–75.8)	69.7 (69.4–70.1)	Reference
	Other	944	57 (37–74)	15.4 (14.4–16.4)	28.1 (26.0–30.3)	0.47 (0.42–0.53)
	Unknown	8,461	69 (50–80)	N/A	N/A	N/A
*Klebsiella* spp.	Asian	4,212	64 (47–76)	15.5 (15.1–16.0)	30.1 (29.1–31.1)	1.58 (1.53–1.63)
	Black	2,237	59 (47–72)	18.8 (18.0–19.6)	32.5 (31.0–34.2)	1.84 (1.76–1.92)
	Mixed	504	54 (27–70)	6.0 (5.5–6.6)	21.9 (19.4–24.6)	1.01 (0.93–1.11)
	White	45,993	73 (61–82)	20.1 (19.9–20.3)	18.7 (18.5–18.9)	Reference
	Other	308	59 (43–71)	5.0 (4.5–5.6)	9.3 (8.1–10.6)	0.51 (0.46–0.57)
	Unknown	2,419	66 (47–78)	N/A	N/A	N/A
*P. aeruginosa*	Asian	1,271	60 (41–72)	4.7 (4.4–4.9)	8.2 (7.7–8.7)	1.18 (1.11–1.25)
	Black	902	57 (43–69)	7.6 (7.1–8.1)	12.4 (11.5–13.4)	1.84 (1.72–1.97)
	Mixed	192	45 (13–63)	2.3 (2.0–2.6)	6.6 (5.3–8.0)	0.91 (0.78–1.04)
	White	18,106	73 (61–83)	7.9 (7.8–8.0)	7.4 (7.3–7.5)	Reference
	Other	103	56 (34–74)	1.7 (1.4–2.0)	3.1 (2.4–3.8)	0.43 (0.35–0.51)
	Unknown	941	67 (50–78)	N/A	N/A	N/A
MSSA	Asian	3,170	50 (21–68)	11.7 (11.3–12.1)	18.0 (17.2–18.7)	0.80 (0.72–0.89)
	Black	1,608	50 (29–62)	13.5 (12.9–14.2)	18.8 (17.7–19.9)	0.88 (0.79–0.99)
	Mixed	575	29 (5–53)	6.9 (6.3–7.5)	13.9 (12.1–15.9)	0.60 (0.52–0.69)
	White	53,071	67 (50–80)	23.2 (23.0–23.4)	22.1 (21.9–22.3)	Reference
	Other	250	45 (18–61)	4.1 (3.6–4.6)	5.6 (4.7–6.4)	0.27 (0.23–0.32)
	Unknown	2,583	59 (37–75)	N/A	N/A	N/A
MRSA	Asian	380	53 (29–70)	1.4 (1.3–1.5)	2.2 (2.0–2.5)	1.79 (1.51–2.10)
	Black	153	51 (33–66)	1.3 (1.1–1.5)	1.9 (1.6–2.3)	1.59 (1.29–1.95)
	Mixed	37	40 (28–55)	0.4 (0.3–0.6)	1.2 (0.6–1.9)	0.89 (0.61–1.26)
	White	3,035	70 (51–82)	1.3 (1.3–1.4)	1.3 (1.2–1.3)	Reference
	Other	23	49 (24–66)	0.4 (0.2–0.6)	0.7 (0.4–1.1)	0.65 (0.41–0.98)
	Unknown	184	58 (37–77)	N/A	N/A	N/A

*E. coli*: *Escherichia coli*; IMD: index of multiple deprivation; MRSA: meticillin-resistant *Staphylococcus aureus*; MSSA: meticillin-susceptible *Staphylococcus aureus*; N/A: not applicable; *P. aeruginosa*: *Pseudomonas aeruginosa*.

^a^ Median age in years with (interquartile range).

^b^ Rates are incidence rates per 100,000 population per year.

^c^ Point estimate (95% confidence interval).

The Mixed and Other EGs had lower crude and age-standardised incidence rates than the White group for *E. coli*, MSSA and MRSA; however, this could be due to poor ethnicity data quality for the former two groups (see Discussion).

Median age at time of infection for bacteraemia overall appeared to be lower in the Black EG (58 years) and Asian EG (64 years) compared with the White EG (74 years); this was particularly marked for MRSA (respectively 51, 53 and 70 years) and MSSA (50, 50 and 67 years).

### Incidence by ethnic subgroup

As illustrated in Supplementary Figure 1, further analysis by ethnic subgroup showed that, within the Asian EG, the Bangladeshi and Pakistani ethnic subgroups appeared to have higher age-standardised incidence of bacteraemia compared with the Indian and Chinese groups (respectively 213.2, 165.6, 127.8 and 100.0 cases per 100,000 population). The Other White subgroup, which includes White ethnic minorities, also seemed to have higher age-standardised incidence compared with the White British majority subgroup and the White Irish subgroup (respectively 169.4, 119.0 and 83.9 cases per 100,000 population). Within the Black EG, the Other Black subgroup appeared to have higher incidence of MSSA, *E. coli*, *Klebsiella* spp. and *P. aeruginosa* bacteraemia, and a similar general pattern was seen with the Other Asian subgroup; however, this could be due to misclassification of ethnic subgroup in the health records (see Discussion).

### Incidence by ethnic group and onset of infection

**Figure 3:**
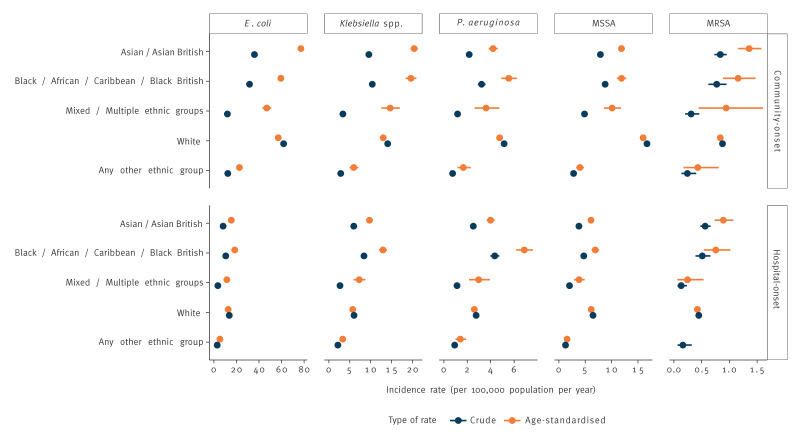
Crude and age-standardised incidence of community- and hospital-onset bacteraemia by ethnic group, England, January 2018–December 2022 (n = 342,787 cases)

Differences in age-stratified incidence rates across ethnic groups were identifiable not only in CO cases but also in HO cases, even if with occasionally differing patterns (Figure 3). This is detailed in Supplementary Table 5.

For all three Gram-negative bacteraemias, the age-standardised HO rate was highest in the Black EG, followed by the Asian and White groups. The age-standardised CO rate of *P. aeruginosa* was also highest in the Black EG, while for *E. coli* it was higher in the Asian group, and for *Klebsiella* spp. it was highest in both.

The age-standardised rate of CO MSSA was higher in the White EG than in the Asian and Black EG; for HO cases, incidence was comparable across these three groups.

For MRSA, age-standardised incidence was higher in the Asian and Black EG for both CO and HO; however, smaller counts led to wide 95% CI.

### Case fatality

Mortality linkage was successful in 338,405 of 342,787 cases (98.7%).

The crude 30-day CFR was comparable across deprivation groups, ranging between 17.6% (95% CI: 17.3–17.9%) in the most deprived quintile to 18.1% (95% CI: 17.8–18.4%) in the least deprived quintile. However, this was influenced by age. After adjusting for age, the estimated odds of dying within 30 days in the most deprived quintile was 15% higher than in the least deprived quintile (aOR: 1.13–1.19).

The unadjusted 30-day CFR was highest in the White ethnic group (18.3%; 95% CI: 18.1–18.4%); it was 14.1% in the Asian (95%: 13.6–14.6%) and 13.9% in the Black EG (95% CI: 13.3–14.6%). After adjusting for age, compared with the White EG, there was no evidence of disproportional odds of dying within 30 days in the Black EG, while the Asian EG had slightly lower odds (aOR: 0.92; 95% CI: 0.89–0.96).

## Discussion

Between 2018 and 2022, we observe disproportionately high age-adjusted incidence of bacteraemia in deprived areas of England and among minority ethnic groups. This constitutes a health equity concern, building on the body of evidence for the presence of social inequalities in a variety of infections and other health outcomes.

The inequality between most and least deprived areas was largest with *S. aureus* bacteraemia, with the age-adjusted incidence 2.68 times higher for MRSA and 2.26 times higher for MSSA. These findings confirm and expand on those from two Danish studies conducted in 1996–2010, which identified an association between *S. aureus* bacteraemia and different metrics of social deprivation: compared with people in the highest income tertile, those in the lowest tertile had 2.77 higher odds of *S. aureus* bacteraemia; after adjusting for comorbidities, this decreased to 1.69 [9,10]. These findings are also in keeping with a study conducted in London in the late 1990s, highlighting a higher risk of postoperative MRSA infection among people living in the most deprived areas [27], and with a 2012 London study that identified an association between household deprivation and MRSA isolates from any body site [28]. In our study, inequality by deprivation was also present for Gram-negative bacteraemia, but to a lesser extent. Age-adjusted mortality was also higher in the 20% most deprived areas of England.

The crude incidence of these infections was highest in the White EG; however, this was considerably influenced by age, as this population has the highest median age, and older age is a known risk factor for bacteraemia [1]. After age adjustment, we note that the Black and Asian EGs were disproportionately affected by bacteraemia from all pathogens, except for community-onset MSSA bacteraemia. The greatest increase in risk among the Asian and Black groups compared with the White EG were seen for MRSA bacteraemia (+ 79% and + 59% respectively) and *Klebsiella* spp. bacteraemia (+ 58% and + 84% respectively); the Black group also had an 84% higher age-adjusted rate of *P. aeruginosa* compared with the White EG. *E. coli* rates were also increased, but to a lesser extent. These differences have been largely consistent throughout the study period, as previously reported [29]. Interestingly, we saw that the incidence of MSSA bacteraemia was highest in the White EG, even after adjusting for age, which contrasts with our MRSA results. We can speculate that this may reflect possible ethnic disparities in resistance patterns of *S. aureus* colonisation, possibly secondary to differential exposure to healthcare environments, or differential antibiotic usage.

We noted these inequalities also existed in HO cases, even though we anticipated that people from different EGs would have comparable exposures to bacteraemia-causing pathogens in hospital, while in the community various environmental and behavioural factors would be influential. However, there are other patient-level factors that would predispose to bacteraemia regardless of patient location, such as comorbidities. Moreover, using a population denominator for HO infections might lead to biased rate estimates, if minority EGs are hospitalised more often than the White group.

Ethnic groups are internally heterogeneous, with higher rates of bacteraemia observed in the Bangladeshi subgroup compared with the Indian subgroup, and in the White Other subgroup compared with the majority White British subgroup.

Cases of bacteraemia in minority ethnic groups and living in more deprived areas tended to be younger on average than those in the White group and those living in richer areas.

The ethnicity results are only partially consistent with two studies from New Zealand and Australia, conducted in 1997 and 2010, respectively, indicating higher incidence of any *S. aureus* bacteraemia among local minority ethnic groups (Māori, Pacific Islanders, indigenous Australians [11,12]); in our study, minority ethnicities only had a higher risk of MRSA bacteraemia, while CO MSSA bacteraemia was found to be disproportionately affecting the White ethnic group.

There are limited studies on health inequalities in bacteraemia; these findings are, however, generally consistent with studies on social inequalities in healthcare-associated infections [5], COVID-19 [6-8], other infectious diseases [30], and other health outcomes [13,17,31] indicating that people from minority ethnic groups and/or with socioeconomic deprivation have higher burden of disease.

Interestingly, the disproportionately high incidence among minority ethnic groups is not reflected in case fatality; in fact, the Asian EG has slightly lower age-adjusted odds of 30-day mortality, compared with the White group. Reasons for this finding are unclear; it is possible that these associations are influenced by factors not available in this study. Therefore, some degree of caution is required in interpretation.

Inequalities in the incidence of bacteraemia across deprivation and ethnic groups are likely to be complex and multifactorial, involving a different distribution of biological, socioeconomic, cultural, and systemic risk factors across these groups. More deprived areas, and minority ethnic groups, have a higher burden of comorbidities that predispose to bacteraemia [32]. Socioeconomic factors such as poverty, low educational attainment, overcrowding and inadequate housing are also associated with a higher risk of infection [30]. The NHS also employs a disproportionate number of staff from ethnic minorities, especially for roles with frequent close contact with patients [33]; we hypothesise that this could potentially increase the risk of colonisation and infection with pathogens such as MRSA, *P. aeruginosa* or *Klebsiella.* Cultural factors, including social mixing, travel destinations and healthcare-seeking behaviour, also influence infection patterns [34]; antibiotic use, hygiene and wound care, and intravenous drug use, may also be influenced by social and cultural forces. Finally, systemic racism, discrimination and bias contribute to health inequalities and may play a role in bacteraemia, for example when people from ethnic minority groups face barriers to healthcare access or receive inequitable healthcare [35].

Study limitations include an area-level deprivation analysis, misclassification of ethnicity, missing data, lack of comorbidity data, limited stratification by sex, and absence of antimicrobial resistance (AMR) results for Gram-negative bacteria. The IMD is assigned at a local area level and has limited specificity and sensitivity for identifying individuals who are employment or income deprived [36]; moreover, we did not conduct separate analyses on each of its constituent deprivation domains. The percentage of ethnicity data agreement between health data sources and the previous Census (2011) was poor for the Mixed EGs (< 67%), Other Asian (< 60%), Other Black (< 16%) and Any other EG (< 15%) [37]; this has likely led to some misclassification and overestimated rates in these ethnic subgroups; we mitigated this issue by conducting our key ethnicity analyses at broad EG level. Also, this complete-case analysis with 4.4% missing ethnicity data has led to a slight underestimate of ethnicity-stratified incidence rates and possible information bias. Comorbidity data were not available; as this is a known risk factor for bacteraemia and it is likely to be unequally distributed among socioeconomic deprivation and ethnic groups [17], it is possible that these results could partially be mediated by disparities in chronic diseases. We standardised ethnicity- and IMD-specific incidence rates by age but not by sex. While sex differences in the overall risk of bacteraemia are well documented (such as higher incidence in males overall) [29], the male-to-female ratio within each ethnic group in the English population is relatively even [3]. As a result, we considered that additional standardisation by sex would only have had a small impact on the estimates of incidence by ethnic group. AMR data for Gram-negative bacteraemia cases are not available in our surveillance platform and would have required additional linkage, which was beyond the scope of this study but may be considered as part of follow-up investigations. Finally, it was not possible to inspect the complex intersection of ethnicity and socioeconomic deprivation with regression models due to the lack of population estimates concurrently stratified by these two factors and age. It is probable that the inequalities by ethnicity are partly explained by deprivation, and vice versa. However, we note that the finding of higher incidence both in deprived areas and ethnic minorities does not map across all pathogens, as the incidence of community-onset MSSA bacteraemia was higher in the White EG. Moreover, other studies have identified worse health outcomes among people from minority ethnicities even after accounting for socioeconomic disadvantage [13,31]; this would indicate that other variables linked to ethnicity, but not socioeconomic disadvantage might have an effect, including the aforementioned systemic, cultural or biological factors. 

## Conclusion

Findings from this study, along with other published data, provide robust evidence that ethnicity and socioeconomic deprivation are associated with a higher risk of acquiring bacteraemia caused by *S. aureus*, *E. coli, Klebsiella* spp. and *P. aeruginosa*; therefore, continual monitoring of metrics of bacteraemia stratified by social factors is a prerequisite for public health efforts in this area to be equitable. The UK Health Security Agency has recently started publishing more analyses of health outcomes stratified by ethnic group and IMD. Further quantitative studies, as well as qualitative research, are required to explore the factors underlying these inequalities to potentially identify measures to close the gap. Furthermore, residents of deprived areas and Black and Asian people should feature as high-risk populations in addition to the generally well-known categories, such as care home residents and pregnant, older, or immunocompromised people, and be included when considering healthcare strategies and interventions.

## Supplementary Data

468000 Supplementary Materials
