# Supplementary material for: Health inequalities in incidence of bacteraemias: a national surveillance and data linkage study, England, 2018 to 2022
Source: Euro Surveill. 2025 Mar 6;30(9):2400312. doi: 10.2807/1560-7917.ES.2025.30.9.2400312 (PMC11887031; doi:10.2807/1560-7917.ES.2025.30.9.2400312)
Supplement: Supplementary Materials [file 2400312_SupplementaryMaterial.pdf]

Supplementary material

This supplementary material is hosted by *Eurosurveillance* as supporting information alongside the article *Health inequalities in incidence of bacteraemias: a national surveillance and data linkage study, England, 2018 to 2022*, on behalf of the authors, who remain responsible for the accuracy and appropriateness of the content. The same standards for ethics, copyright, attributions and permissions as for the article apply. Supplements are not edited by *Eurosurveillance,* and the journal is not responsible for the maintenance of any links or email addresses provided therein.

Supplementary Table 1. Summary of combined *S. aureus* bacteraemia cases identified, England, January 2018 - December 2022 (n = 65,069 cases).

**Characteristic**	**Group**	Number	**%**
Phenotype	MSSA	61,257	94.1%
	MRSA	3,812	5.9%
Age	[b]	65	47-79
Sex	Female	23,561	36.2%
	Male	41,464	63.8%
	Unknown	44	N/A
Index of Multiple Deprivation quintile	1st (most deprived)	16,769	26.3%
	2nd	13,613	21.3%
	3rd	12,284	19.3%
	4th	11,155	17.5%
	5th (least deprived)	9,942	15.6%
	Unknown	1,306	N/A
Ethnic group	Asian	3,550	5.7%
	Black	1,761	2.8%
	Mixed	612	1.0%
	White	56,106	90.1%
	Other	273	0.4%
	Unknown	2,767	N/A
Onset	Community-onset	46,331	71.2%
	Hospital-onset	18,738	28.8%

[a] Percentages refer to the total of known values in each column.

[b] Age is expressed in years and is summarised as median and interquartile range.

N/A: not applicable.

Supplementary Table 2. Combined *S. aureus* bacteraemia by Index of Multiple Deprivation (IMD) quintile, England, January 2018 - December 2022 (n = 65,069 cases).

IMD quintile	Cases	Crude rate [a]	Age-standardised rate [a]	Age-adjusted rate ratio [a]
**1st (most deprived)**	16,769	29.7 (29.3, 30.2)	35.3 (34.8, 35.9)	2.29 (2.06, 2.54)
**2nd**	13,613	23.5 (23.1, 23.9)	26.1 (25.7, 26.6)	1.63 (1.47, 1.82)
**3rd**	12,284	21.5 (21.1, 21.8)	21.4 (21.0, 21.8)	1.34 (1.20, 1.49)
**4th**	11,155	20.0 (19.7, 20.4)	18.6 (18.3, 19.0)	1.16 (1.04, 1.29)
**5th (least deprived)**	9,942	18.2 (17.8, 18.6)	16.2 (15.9, 16.6)	(Reference)
**Unknown**	1,306	N/A	N/A	N/A

[a] Point estimate (95% confidence interval). N/A: not applicable.

Supplementary Table 3. Combined *S. aureus* bacteraemia by ethnic group, England, January 2018 - December 2022 (n = 65,069 cases).

Ethnic group	Cases	Crude rate [a]	Age-standardised rate [a]	Age-adjusted rate ratio [a]
**Asian**	3,550	13.1 (12.7, 13.5)	0.6 (0.4, 0.7)	0.85 (0.76, 0.95)
**Black**	1,761	14.8 (14.1, 15.5)	1.3 (1.0, 1.6)	0.92 (0.82, 1.03)
**Mixed**	612	7.3 (6.8, 7.9)	4.0 (2.9, 5.2)	0.61 (0.53, 0.70)
**White**	56,106	24.5 (24.3, 24.7)	0.0 (0.0, 0.1)	(Reference)
**Other**	273	4.4 (3.9, 5.0)	2.6 (2.0, 3.3)	0.29 (0.24, 0.33)
**Unknown**	2,767	N/A	N/A	N/A

[a] Point estimate (95% confidence interval). N/A: not applicable.

Supplementary Table 4. All bacteraemia cases by ethnic group and Index of Multiple Deprivation quintile, England, January 2018 to December 2022 (n = 342,787 cases).

	**Asian**, N = 21,025		**Black**, N = 9,924		**Mixed**, N = 2,610		**White**, N = 293,012		**Other**, N = 1,628		**Unknown**, N = 14,588	
	n	% [a]	n	%	n	%	n	%	n	%	n	%
**1st (most deprived)**	6,581	31.4%	3,804	38.5%	817	31.6%	63,581	21.8%	506	31.4%	2,288	20.8%
**2nd**	6,038	28.8%	3,359	34.0%	614	23.7%	58,362	20.0%	436	27.0%	2,263	20.6%
**3rd**	3,933	18.8%	1,538	15.6%	519	20.1%	58,990	20.3%	301	18.7%	2,286	20.8%
**4th**	2,541	12.1%	733	7.4%	345	13.3%	57,564	19.8%	206	12.8%	2,103	19.2%
**5th (least deprived)**	1,849	8.8%	446	4.5%	293	11.3%	52,614	18.1%	163	10.1%	2,040	18.6%
**Unknown IMD**	83	N/A	44	N/A	22	N/A	1,901	N/A	16	N/A	3,608	N/A

[a] Percentages refer to the total in each ethnic group.

Supplementary Figure 1. Crude and age-standardised incidence rate of bacteraemia by ethnic subgroup, England, January 2018 to December 2022 (n = 342,787 cases).

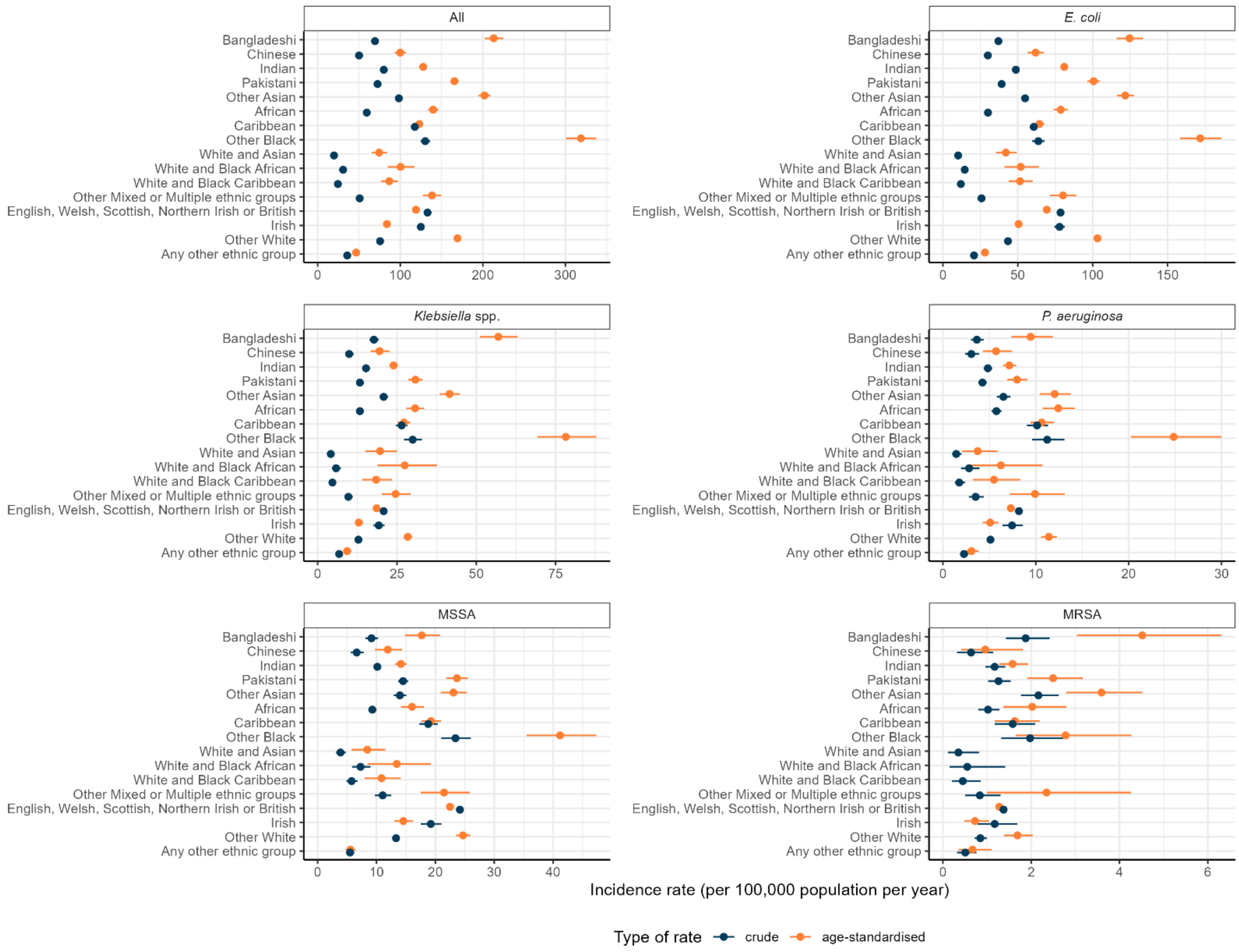

Supplementary Table 5. Age-standardised rates of bacteraemias by ethnic group and infection onset, England, January 2018 to December 2022 (n = 342,787 cases).

Pathogen	Ethnic group	Community-onset age-standardised rate[1]	Hospital-onset age-standardised rate[1]
E. coli	Asian	77.1 (75.4, 78.8)	15.5 (14.7, 16.2)
	Black	59.3 (57.2, 61.6)	18.5 (17.3, 19.7)
	Mixed	46.9 (43.1, 50.8)	11.5 (9.8, 13.5)
	White	57.0 (56.7, 57.3)	12.7 (12.6, 12.9)
	Other	22.7 (20.8, 24.7)	5.4 (4.5, 6.4)
Klebsiella spp.	Asian	20.4 (19.5, 21.3)	9.7 (9.2, 10.3)
	Black	19.6 (18.4, 20.9)	12.9 (12.0, 13.9)
	Mixed	14.6 (12.5, 17.0)	7.3 (5.9, 8.7)
	White	13.0 (12.9, 13.2)	5.7 (5.6, 5.8)
	Other	6.0 (5.0, 7.1)	3.3 (2.7, 4.1)
P. aeruginosa	Asian	4.2 (3.8, 4.6)	4.0 (3.7, 4.3)
	Black	5.5 (4.9, 6.2)	6.9 (6.2, 7.6)
	Mixed	3.6 (2.6, 4.7)	3.0 (2.2, 3.9)
	White	4.8 (4.7, 4.9)	2.6 (2.5, 2.7)
	Other	1.7 (1.2, 2.3)	1.4 (1.0, 1.9)
MSSA	Asian	11.9 (11.3, 12.5)	6.1 (5.7, 6.5)
	Black	11.9 (11.0, 12.8)	6.9 (6.2, 7.6)
	Mixed	10.1 (8.5, 11.8)	3.8 (2.9, 4.9)
	White	16.0 (15.8, 16.1)	6.1 (6.0, 6.2)
	Other	4.0 (3.3, 4.8)	1.6 (1.2, 2.0)
MRSA	Asian	1.4 (1.2, 1.6)	0.9 (0.7, 1.1)
	Black	1.2 (0.9, 1.5)	0.8 (0.5, 1.0)
	Mixed	0.9 (0.4, 1.6)	0.2 (0.1, 0.5)
	White	0.8 (0.8, 0.9)	0.4 (0.4, 0.5)
	Other	0.4 (0.2, 0.8)	-

[1] Point estimate (95% confidence interval), per 100,000 population per year
